# The Impeding Role of Self-Critical Perfectionism on Therapeutic Alliance During Treatment and Eating Disorder Symptoms at Follow-up in Patients with an Eating Disorder

**DOI:** 10.5334/pb.297

**Published:** 2016-04-15

**Authors:** Jolene van der Kaap-Deeder, Jos Smets, Liesbet Boone

**Affiliations:** 1 Department of Developmental, Social, and Personality Psychology, Ghent University, Ghent, Belgium; 2 Psychiatric nurse, Alexian Brothers Psychiatric Hospital (Unit Ter Berken), Tienen, Belgium

**Keywords:** Self-criticism, therapeutic alliance, eating disorder patients, therapeutic change

## Abstract

This study examines the impeding role of self-critical perfectionism at onset of treatment on therapeutic alliance during treatment and eating disorder symptoms at follow-up in patients with an eating disorder. Participants were 53 female patients with a mean age of 21.1 years treated for an eating disorder in a specialized inpatient treatment unit. Self-critical perfectionism was assessed at admission, therapeutic alliance was assessed during treatment (after three months of treatment), and eating disorder symptoms were assessed at admission, after three months and one year later. Self-critical perfectionism negatively related to treatment alliance with the therapist. Although self-critical perfectionism was not directly predictive of subsequent changes in eating disorder symptoms, it was indirectly related to less reduction in body dissatisfaction through the therapeutic alliance. These results point to the importance of self-critical perfectionism in the therapeutic alliance and in changes in body image problems. Treatment implications are discussed.

Many studies have shown that patients’ self-critical (SC) perfectionism interferes with the treatment of depression (Blatt & Zuroff, 2005), as it impedes therapeutic alliance (Shahar, Blatt, Zuroff, Krupnick, & Sotsky, 2004) and therapeutic progress (Zuroff et al., 2000). Patients with high levels of self-criticism are characterized by a negative representation of the self as they engage in constant and harsh self-scrutiny and self-evaluation, and have a negative representation of others as they are chronically concerned about others’ criticism or evaluation (Blatt, 2008; Dunkley & Kyparissis, 2008). Although it has not been directly tested, it has been suggested (e.g., Shahar et al., 2004) that the difficulties that individuals with high levels of SC perfectionism experience in engaging in a therapeutic alliance, might be because they project their own self-criticism onto the therapist and might be less likely to trust the therapist and to disclose themselves in treatment.

SC perfectionism has been found to be not only associated with increased vulnerability for depressive symptoms, but also with the development of eating disorder (ED) symptoms (Egan, Wade, & Shafran, 2011). Cross-sectional (e.g., Ferreira, Pinto-Gouveia, & Duarte, 2013), longitudinal (e.g., Mackinnon et al., 2011), and experimental research (e.g., Boone, Soenens, Vansteenkiste, & Braet, 2012) in both clinical and non-clinical samples have shown that individuals with high levels of SC perfectionism have an increased risk to experience a wide range of ED symptoms, such as binge eating, dieting, and body dissatisfaction. To illustrate, a study of Fennig and colleagues (2008) showed that self-criticism emerged as a strong predictor of ED symptoms in inpatient adolescent females. Additionally, Kelly, Carter, Zuroff, and Borairi (2013) showed that experiencing low self-compassion and high fear of self-compassion (which can be seen as important aspects of SC perfectionism) at the start of treatment resulted in poorer treatment responses (i.e., no significant change in ED symptoms and shame over a 12-week period) in a group of patients with an ED.

Although, to date, there is ample evidence that patients’ high levels of SC perfectionism interferes with a) therapeutic progress and b) the development of a good therapeutic alliance in the treatment of depression, no research has investigated this in patients with an ED, nor has it been tested with a longitudinal design. Yet, this seems important as patients with an ED often display high levels of SC perfectionism (Egan et al., 2011) and the therapeutic alliance has been shown to be predictive for therapy outcomes (Martin, Garske, & Davis, 2000), also in EDs (Brown, Mountford, & Waller, 2013). Therefore, the aim of this study was twofold. First, we aimed to examine the effect of SC perfectionism at admission on subsequent changes in ED symptoms. Second, we aimed to test a mediating model in which therapeutic alliance during treatment mediates the relation between patients’ SC perfectionism at onset of treatment and changes in ED symptoms over time.

## Method

### Participants and Procedure

Participants were 53 female patients (age range = 14.6–44.3; *M* = 21.1; *SD* = 5.5) treated for an ED in a specialized inpatient treatment unit Alexian Brothers Psychiatric Hospital. The mean duration of the ED was 4,5 years (range = 0.5–15). Based on a diagnostic interview by an experienced psychiatrist conducted at intake, 25 (47%) were diagnosed with anorexia nervosa-restrictive type, 6 (11%) with anorexia nervosa-purging type, 7 (14%) with bulimia nervosa, and 15 (28%) with eating disorder not otherwise specified. Patients were free to participate in the study and those who agreed to participate filled out an informed assent. Parental permission was obtained for patients younger than 18 years. The study procedure was approved by the ethical committee of Ghent University Hospital and the local ethical committee of the ED clinic.

Patients filled out questionnaires at admission (T1), after 3 months of treatment (T2), and at follow-up one year after the start of treatment (T3). Of the 97 patients who completed questionnaires at T1, only 53 also filled out questionnaires at T2, and of this subsample 21 patients also completed the questionnaires at T3 (see Table 1 for a flow chart). Main reasons for drop-out were discharge from treatment and refusal to participate at the follow-up assessments. A binary logistic regression analysis showed that the patients who only participated at T1 (n = 97) did not differ from the 53 patients (T1 & T2) [χ²(4) = 3.74, *p* > .05] nor from the 21 patients (T1, T2, & T3) [χ²(4) = 7.30, *p* > .05] on background variables such as age, BMI at admission, diagnosis and duration of illness, nor on study variables SC perfectionism and ED symptoms at T1 compared to the 53 patients [χ²(4) = 2.29, *p* > .05] and 21 patients [χ²(4) = 3.1, *p* > .05]. In addition for those who participated at T1 and T2, participants with (n = 21) and without (n = 32) complete data at T3 were compared using Little’s Missing Completely at Random (MCAR) test (Little, 1988). This test revealed that data were missing completely at random (χ²(1871) = 32.35, *p* = 1.00; χ²/*df* ratio = .02). Therefore, for the 53 patients who participated at T1 and T2, missing values at T3 were estimated using Expectation Maximization algorithm (EM) in SPSS 21.

**Table 1:** Flow Chart of Sample Size and Drop-out per Measurement. Patients who participated at T1 and T2 were selected (n = 53), missing data for T3 were estimated.

Number of participants at Time 1		97
Drop-out	44	
Number of participants at Time 2		53
Drop-out	32	
Number of participants at Time 3		21

### Measures

**Self-critical Perfectionism (T1).** SC perfectionism was measured using the Multidimensional Perfectionism Scale of Frost and colleagues (F-MPS; Frost, Marten, Lahart, & Rosenblate, 1990). Items were answered on a 5-point Likert scale. The subscales Concern over Mistakes (9 items; α = .93; e.g., “People will think less of me if I make a mistake”) and Doubts about Actions (4 items; α = .72; e.g., “It takes me a long time to do something right”) were combined to assess SC perfectionism (α = .92). The subscale Personal Standards perfectionism (7 items; α = .90; e.g., “I set higher goals for myself than most other people”) was used to asses Personal Standards (PS) perfectionism. In line with previous studies (Soenens et al., 2008), scores for SC perfectionism were adjusted for scores of PS perfectionism in order to really tap into a self-critical attitude instead of the setting of high personal standards. This was deemed necessary because both perfectionism components have been shown to be intertwined (Boone, Soenens, Mouratidis, Vansteenkiste, & Verstuyf, 2012). Therefore, a residual score was computed by regressing SC perfectionism on the subscale personal standards of the F-MPS (see Soenens et al., 2008).

**Therapeutic Alliance (T2).** The short form (12-items) of the patient-reported Working Alliance Inventory (WAI-SR) (Tracey & Kokotovic, 1989) was used to assess the therapeutic alliance (5 items; e.g., “I feel that my therapist appreciates me”). For the purpose of the study, we did not use the other subscales of the WAI-SR, which assess patients’ opinion of the shared goals (3 items) and tasks of treatment (4 items). The scale ranged from 1 to 5, where higher scores indicate a stronger therapeutic alliance. Cronbach’s alpha was .81 in this study.

**Eating Disorder Symptomatology (T1, T2, T3).** The well-validated eating disorder inventory II (EDI-II; Garner, 1991) is one of the most widely used standardized self-report questionnaires assessing psychological and behavioral characteristics associated with EDs, such as anorexia nervosa (AN) and bulimia nervosa (BN). For the purpose of this study, we used the subscales Drive for Thinness (DT; 7 items; e.g., “I’m terribly scared to gain weight”), Bulimia (BUL; 7 items; e.g., “I have episodes of eating in which I feel like I cannot stop eating“), and Body Dissatisfaction (BD; 9 items; e.g., “I think my hips are too big”). Responses were rated on a 6-point scale ranging from ‘never’ to ‘always’. In this study, all subscales were highly reliable with Cronbach’s alpha’s of .88, .94, and .91 at T1, and .88, .94, and .91 at T2, and .93, .86, and .94 at T3 for DT, BUL, and BD respectively.

**Depressive Symptoms (T1).** To assess depressive symptoms, the subscale Depressive Symptoms (16 items; e.g., “I feel lonely“) of the Dutch version of the well-validated Symptom Checklist 90-Revised (Arrindell & Ettema, 1986) was used. In this study, the subscale had a good reliability with a Cronbach’s alpha of .89.

### Analytic Plan

One-way ANOVA’s were performed to test whether mean-levels of study variables differed across diagnostic groups. *T*-tests for dependent samples were used to examine changes in symptoms from admission to follow-up. Zero-order correlation analyses were conducted to examine relations among study variables.

In line with previous studies (e.g., Barber, Connolly, Crits-Christoph, Gladis, & Siqueland, 2000; Freeley, DeRubeis, & Gelfand, 1999), we calculated two symptom change scores. First, a residualized score was calculated in which symptom levels at T2 were regressed on symptom levels at T1, to which we will refere as ‘prior ED-symptom change’. Similarly, a residualized score was calculated in which symptom levels at T3 were regressed on symptom levels at T2, and to which we will refere as ‘subsequent ED-symptom change’. These change scores take into account temporal precedence, and allow to determine whether therapeutic alliance assessed at T2 was releated to prior change in ED symptoms, or to subsequent change in ED symptoms.

For the main analyses, three separate regression analyses were performed in which it was examined whether SC perfectionism T1 predicted a) subsequent change in ED symptoms and b) therapeutic alliance at T2, controlling for duration of illness, BMI at T1, depressive symptoms at T1, and prior ED-symptom change. To examine the mediating effect of therapeutic alliance in the relation between SC perfectionism and subsequent change in ED symptoms, two mediation models were tested with subsequent change in drive for thinness as an outcome in the first model and subsequent change in body dissatisfaction as an outcome in the second model. In these analyses, we followed the four step procedure of Kenny, Kashy, and Bogler (1998), and we controlled for BMI at T1, duration of illness depressive symptomatology at T1, and prior ED-symptom change.

## Results

### Preliminary Analyses

One-way ANOVA’s revealed that there were no differences between diagnostic categories in terms of SC perfectionism at T1 [*F*(3,49) = .58, *p* > .05], drive for thinness at T1 [*F*(3,49) = .56, *p* > .05], T2 [*F*(3,49) = .22, *p* > .05] and T3 [*F*(3,49) = .67, *p* > .05], body dissatisfaction at T1 [*F*(3,49) = 1.76, *p* > .05], T2 [*F*(3,49) = .29, *p* > .05] and T3 [*F*(3,49) = .47, *p* > .05], and therapeutic alliance at T2 [*F*(3,49) = .47, *p* > .05]. However, patients with bulimia nervosa reported significantly more bulimic symptoms compared to the other diagnostic groups [*F*(3,49) = 23.21, *p* < .001 at T1; *F*(3,49) = 16.84, *p* < .001 at T3; *F*(3,49) = 5.16, *p* < .01 at T3]. Given that diagnostic differences were not the main study goal and the small number of patients per diagnostic category, which limits the power, bulimic symptoms will not be further examined.

Both body dissatisfaction and drive for thinness decreased significantly [*t*(52) = 8.65, *p* < .001, Cohen’s *d* = 1.44; *t*(52) = 8.97, *p* < .001, Cohen’s *d* = 1.52, respectively] from T1 to T3 (see Table 2 for the means and *SD*’s). As can be seen in Table 2, correlations showed that SC perfectionism T1 was positively significantly related to body dissatisfaction and depressive symptoms T1, negatively to therapeutic alliance T2, and marginally significant to drive for thinness T2 and body dissatisfaction T3. Therapeutic alliance T2 was only negatively significant related to body dissatisfaction T3, suggesting that the therapeutic alliance might particularly be important for body image improvement at follow-up and not at the beginning of or during treatment. Correlations between SC perfectionism and therapeutic alliance and prior and subsequent ED-symptom change revealed that only subsequent change in body dissatisfaction was significantly related to therapeutic alliance T2 (β = –.79, *p* < .05).^1^

**Table 2:** Means, Standard Deviations, and Correlations between Study Variables.

	1	2	3	4	5	6	7	8	9

1. SC perfectionism T1									
2. DT T1	.22								
3. BD T1	.28*	.77***							
4. Depressive symptoms	.33*	.37**	.34*						
5. Therapeutic alliance T2	−.37**	−.12	−.15	−.31*					
6. DT T2	.25†	.56***	.49***	.13	−.16				
7. BD T2	.22	.33*	.40**	.09	−.18	.74***			
8. DT T3	.09	.25	.12	−.01	−.14	.36**	.30*		
9. BD T3	.23^†^	.27	.29*	.05	−.32*	.35*	.37**	.56***	
Mean	.	5.08	4.93	3.38	3.23	4.14	4.54	3.82	3.79
*SD*	.	.95	.96	.76	.74	1.20	.98	.69	.57

*Note*. Because SC perfectionism is a residual score, mean and standard deviation are not provided. SC = self-critical; DT = drive for thinness; BD = body dissatisfaction. ^†^*p* < .10. *p < .05. **p < .01. ***p < .001.

### Regression Analyses

As can be seen in Table 3, regression analyses showed that, while controlling for BMI at T1, duration of illness, prior ED-symptom change, and depressive symptomatology at T1, SC perfectionism T1 was marginally predictive of therapeutic alliance at T2 (β = –.28, *p* = .053). SC perfectionism T1 was not predictive (*p* > .10) of changes in ED symptoms from T2 to T3.

**Table 3:** Standardized Beta-coefficients of Hierarchical Regression Analyses.

Predictor	Subsequent change in drive for thinness		Subsequent change in body dissatisfaction		Therapeutic alliance T2	

	**β**	***ΔR*²**	**β**	***ΔR*²**	**β**	***ΔR*²**

Step 1		.08		.01		.07
Duration eating disorder	−.27		−.08		−.16	
Body mass index T1	−.02		.01		.23	
Step 2		.00		.00		.09
Prior drive for thinness change	−.01		.		−.04	
Prior body dissatisfaction change	.		−.06		−.11	
Depressive symptoms T1	−.04		.02		−.28*	
Step 3		.00		.03		.07(*)
SC perfectionism T1	−.02		.19		−.28(*)	

*Note*. SC = self-critical; Subsequent change = symptom level change from T2 to T3. (*) *p* = .053. *p < .05.

**Figure 1:**
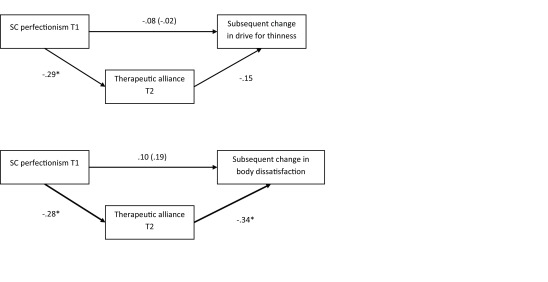
The Mediating Role of Therapeutic Alliance in the Relation Between Self-critical Perfectionism and Subsequent Changes in Body Dissatisfaction and Drive for Thinness. *Note*: Coefficients reported between brackets represent the direct relation between SC perfectionism T1 and the outcome without therapeutic alliance T2 in the model. SC = Self-critical. *p < .05.

Follow-up regression analyses showed that, although SC perfectionism T1 was not directly related to subsequent changes in drive for thinness nor body dissatisfaction, the therapeutic alliance at T2 was a significant intervening variable in the relation between SC perfectionism T1 and decreases in body dissatisfaction T3 (see Figure 1). Bootstrap analysis using bias-corrected and accelerated (BCa) confidence intervals (95%) showed that the indirect effect was significant [.0072; .3267].

## Discussion

This study was the first to show the impeding impact of patients’ SC perfectionism on therapeutic alliance during treatment in patients with an ED. Furthermore, it was shown that, although there was no direct relationship between SC perfectionism and changes in ED symptoms, the difficulties in engaging in a high-quality therapeutic relationship impede improvement in body dissatisfaction for patients with high levels of SC perfectionism. It is important to note that these relations remained true above and beyond the effects of depressive symptoms at T1 and prior ED-symptom change. Our findings are in line with the depression literature, in which the effect of SC perfectionism on therapy outcomes was found to be mediated by an impaired therapeutic alliance (e.g., Shahar et al., 2004; Zuroff et al., 2000) and meta-analyses in which it was shown that therapeutic working alliance is related to therapeutic outcome (Brown et al., 2013; Martin et al., 2000). Surprisingly, although the link has been shown with treatment progress for depressive symptoms (Zuroff et al., 2000), we did not find a direct association between SC perfectionism at T1 and subsequent change in ED symptoms. Potentially, this might be due to our small sample size. Future research may want to further investigate this unexpected finding.

Although this study is innovative and has several strengths, such as a longitudinal study design, there were also some limitations. First, although drop-out was random, there was a relatively high drop-out rate. However, the established relationships were of similar magnitude in the part of the subsample that completed all measures (n = 21) compared to the sample used in this study (n = 53). Second, the heterogeneous and small sample size limited the power and generalizability of the findings. For instance, although some path coefficients were rather large, they appeared to be only marginally significant. Furthermore, due to the small sample size, we could not examine whether there would be differences between diagnostic groups with regard to the intervening role of therapeutic alliance. Although no mean differences in self-criticism or therapeutic alliance between the diagnostic groups were found in this study, it could be that, because patients with anorexia nervosa are highly self-critical and often deny their eating problem (Couturier & Lock, 2006), the model would more strongly apply to patients with anorexia nervosa compared to patients with another ED diagnosis. Possibly, for patients with anorexia nervosa, the relation between SC perfectionism T1 and subsequent change in ED symptoms could have been significant. Third, although it was a strength that we considered temporal precedence by controlling for prior ED-symptom change and examining the effect of therapeutic alliance on subsequent ED-symptom change, it is a limitation that we measured therapeutic alliance at T2 only. Similarly, although SC perfectionism is considered to be a relatively stable personality characteristic (Rice & Aldea, 2006), there is variability within persons (Boone et al., 2012). Therefore, it would have been interesting to control for SC perfectionism at T2 and T3. Future research may want to test the model using a larger sample size and including the variables at all time points using cross-lagged analysis.

Despite these study limitations, our study findings clearly point to the important role of SC perfectionism and the therapeutic alliance in ED treatment, since both either directly or indirectly relate to the course of body image concerns over time. In treatment, SC perfectionism could be targeted using techniques from diverse psychotherapeutic theories (see Kannan & Levitt, 2013). For instance, in cognitive therapy, the focus lays on the modification of early maladaptive schemas that are supposed to underlie self-critical states, through techniques such as cognitive restructuring and reattribution (Boone, Braet, Vandereycken, & Claes, 2013).

Although more research is needed, Acceptance and Commitment Therapy (ACT), which has been described as the third wave of cognitive behavioral psychotherapy, seems to hold promise for the treatment of EDs, and anorexia nervosa in particular (Berman, Boutelle, & Crow, 2009). One of the key components of ACT is the development of an increased psychological flexibility. Increasing psychological flexibility tries to decrease the avoidant, rigid, and escape-avoidance coping styles of the patient (e.g., self-criticism). At the same time, such an approach will foster openness towards engaging in a good therapeutic alliance and towards change-facilitative experiences (Wollburg, Meyer, Osen, & Lowe, 2013), which in time will reduce symptomatology.

Also, Compassion Focused Therapy has been shown to be effective in reducing self-criticism. For instance, in a pilot study it was shown that 12 two-hour sessions of compassionate mind training was effective in reducing self-criticism (and depression, anxiety, shame, inferiority and submissive behavior) in six patients (Gilbert & Procter, 2006). The main goal of this treatment is to learn the patient to be more compassionate for the self and to soothe themselves and as such counter their self-criticism (Barnard & Curry, 2011). Besides this, therapists treating patients with an ED should pay attention to the therapeutic alliance throughout the treatment process, especially for those patients high in self-criticism. Therapists should be aware of the possibility that SC perfectionistic patients project their own self-criticism onto the therapist, and if appropriate, the therapist may try to bring this into the transference (Andersen & Miranda, 2000).

## Conclusion

This study showed that the intrapersonal characteristic of SC perfectionism may impede the quality of or the ability to engage in a positive relation with the therapist. Furthermore, it was found that a less beneficial therapeutic alliance impeded treatment progress for body image problems in patients with an ED. Therefore, SC perfectionism should be targeted in ED treatment as it may act as a maintaining factor for body dissatisfaction.

## Competing Interests

The authors declare that they have no competing interests.

## Footnotes

Attesting to the robustness of our findings, additional correlational analyses (see Table A1 of the Appendix) showed that the reported magnitude and direction of effects as obtained with our study sample (n = 53) were similar for those participants who participated at all three measurement waves (n = 21).

## Appendix

**Table A1:** Means, Standard Deviations, and Correlations between Study Variables for Patients Participating at all Measurement Waves (n = 21).

	1	2	3	4	5	6	7	8	9

1. SC perfectionism T1									
2. DT T1	.52*								
3. BD T1	.65**	.83***							
4. Depressive symptoms	.54*	.50*	.45*						
5. Therapeutic alliance T2	–.44*	–.37	–.38	–.36					
6. DT T2	.42^†^	.57**	.47*	.06	–.25				
7. BD T2	.47*	.20	.28	.03	–.16	.77***			
8. DT T3	.19	.35	.24	-.01	–.30	.60**	.49*		
9. BD T3	.43^†^	.40	.58*	.03	–.60**	.61*	.57*	.55*	

*Note*. SC = self-critical; DT = drive for thinness; BD = body dissatisfaction. ^†^*p* < .10. **p* < .05. ***p* < .01. ****p* < .001.
